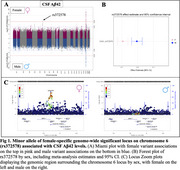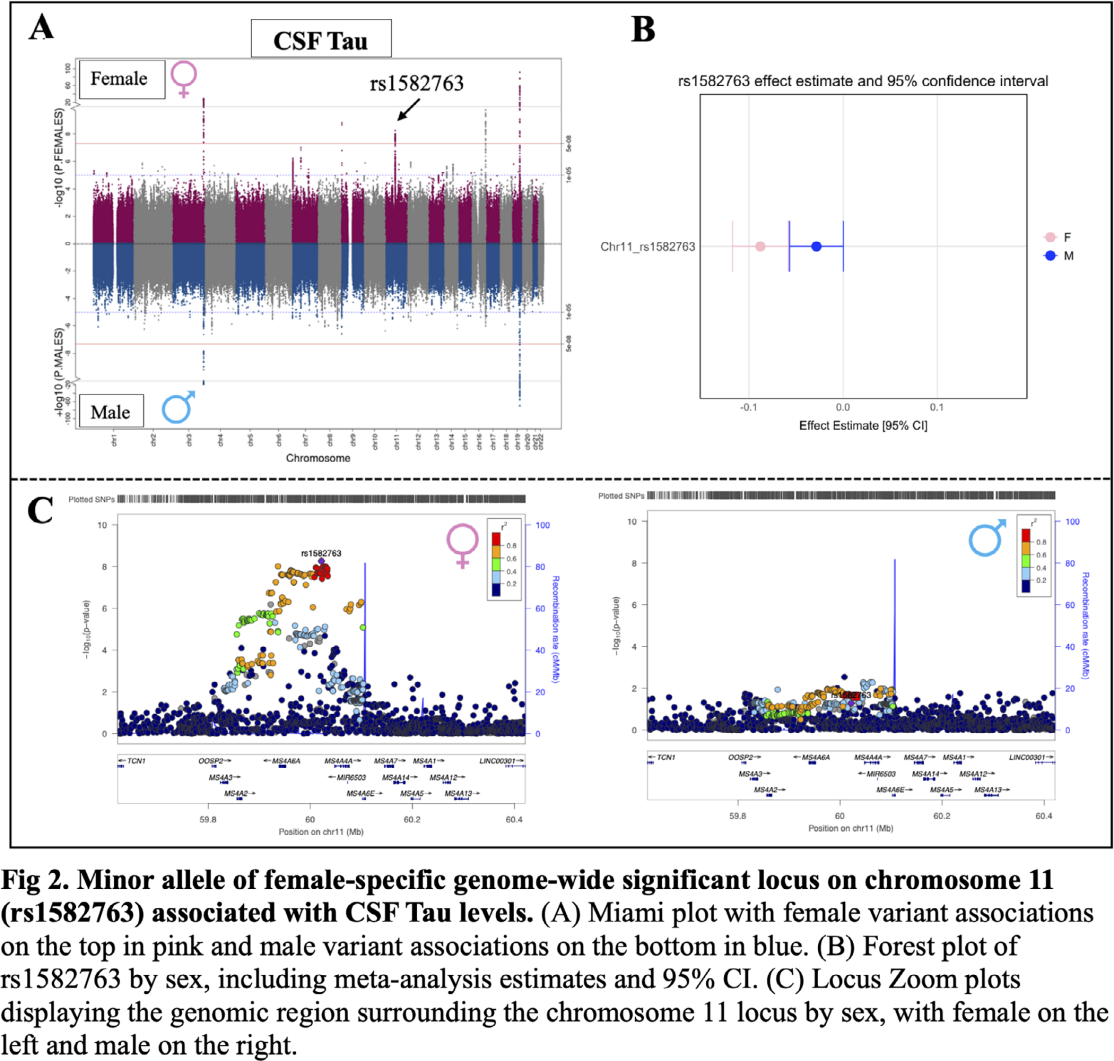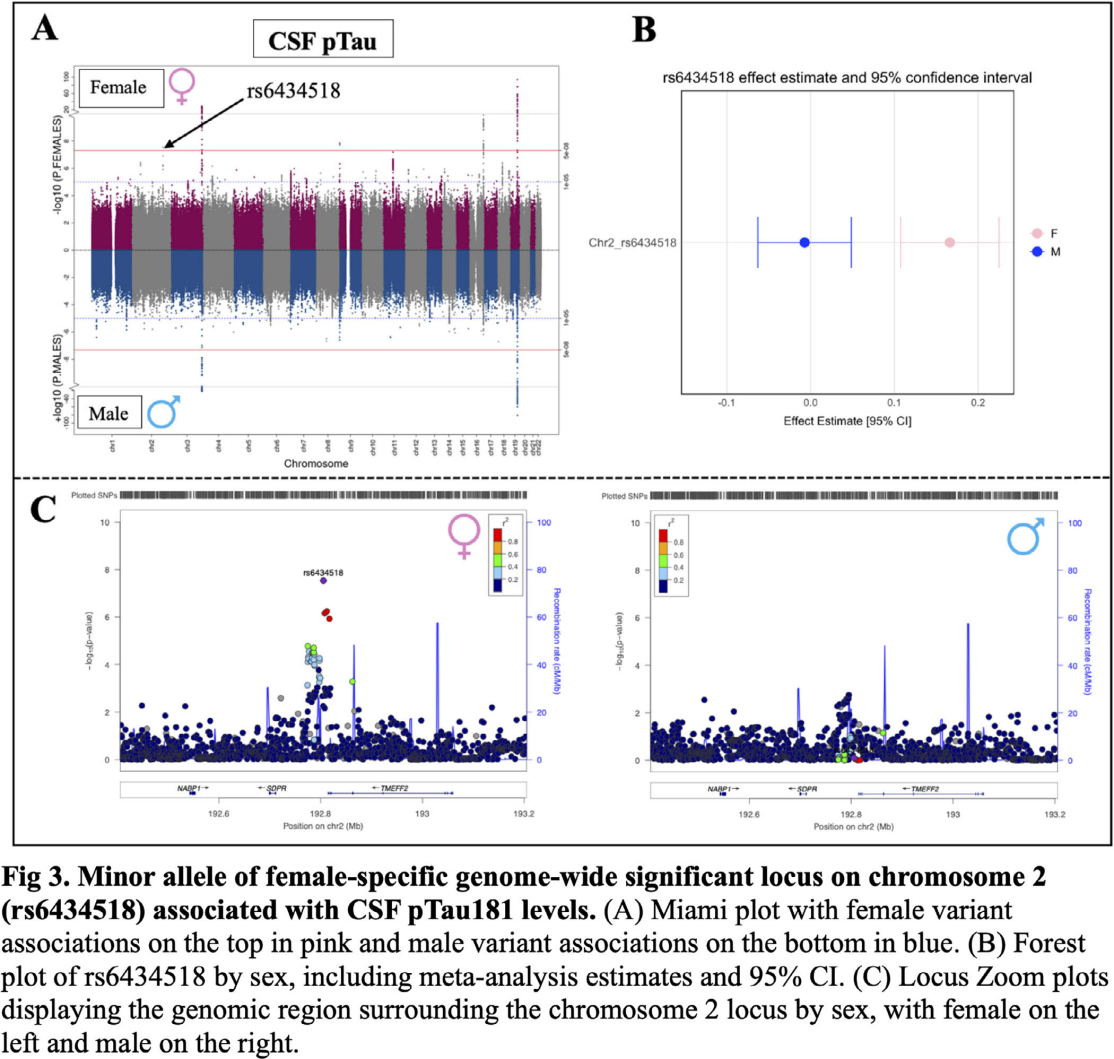# Sex‐stratified GWAS meta‐analyses reveal novel sex‐specific association with CSF biomarkers of Alzheimer's Disease

**DOI:** 10.1002/alz70855_102560

**Published:** 2025-12-23

**Authors:** Ting‐Chen Wang, Jigyasha Timsina, Chenyang Jiang, Daniel L McCartney, Feifei Tao, Federica Anastasi, Patricia Genius, Blanca Rodríguez‐Fernández, Arcadi Navarro, Raquel Puerta, Sven J van der Lee, Riccardo E Marioni, Lars Bertram, Nagle W. Michael, Rebecca Sims, Natalia Vilor‐Tejedor, Joseph Bradley, Muhammad Ali, Ciyang Wang, Menghan Liu, Agustin Ruiz, Maria Victoria Fernandez, Julie Williams, John P. Budde, Betty Tijms, Atahualpa Castillo, Kaj Blennow, Henrik Zetterberg, Alberto Lleo, Virginia M. M.‐Y. Lee, Amanda J Heslegrave, Pau Pastor, Elaine R. Peskind, Andrew J. Saykin, John C. Morris, Suzanne E. Schindler, David M. Holtzman, Matthias Riemenschneider, Marilyn S. S. Albert, Vivianna M Van Deerlin, Leslie M. Shaw, Yun Ju Ju Sung, Timothy J. Hohman, Carlos Cruchaga, Logan Dumitrescu

**Affiliations:** ^1^ Vanderbilt Genetics Institute, Vanderbilt University Medical Center, Nashville, TN, USA; ^2^ Vanderbilt Memory & Alzheimer's Center, Vanderbilt University Medical Center, Nashville, TN, USA; ^3^ NeuroGenomics and Informatics Center, Washington University School of Medicine, St. Louis, MO, USA; ^4^ Department of Psychiatry, Washington University School of Medicine, St. Louis, MO, USA; ^5^ Alzheimer Center Amsterdam, Neurology, Vrije Universiteit Amsterdam, Amsterdam UMC location VUmc, Amsterdam, Netherlands; ^6^ Amsterdam Neuroscience, Neurodegeneration, Amsterdam, Netherlands; ^7^ Section Genomics of Neurodegenerative Diseases and Aging, Department of Human Genetics, Vrije Universiteit Amsterdam, Amsterdam, Noord‐Holland, Netherlands; ^8^ University of Edinburgh, Edinburgh, United Kingdom; ^9^ Human Biology Integration Foundation, Genetics‐Guided Dementia Discovery, Eisai Inc, Cambridge, MA, USA; ^10^ Barcelonaβeta Brain Research Center (BBRC), Pasqual Maragall Foundation, Barcelona, Spain; ^11^ Centre for Genomic Regulation (CRG), Barcelona Institute of Science and Technology (BIST), Barcelona, Spain; ^12^ BarcelonaBeta Brain Research Center (BBRC), Barcelona, Spain; ^13^ Research Center and Memory Clinic, Fundació ACE Institut Català de Neurociències Aplicades ‐ Universitat Internacional de Catalunya (UIC), Barcelona, Spain; ^14^ University of Lübeck, Lübeck, Germany; ^15^ Division of Psychological Medicine and Clinical Neurosciences, Cardiff University, Cardiff, United Kingdom; ^16^ Radboud University Medical Center, Nijmegen, Netherlands; ^17^ Ace Alzheimer Center Barcelona – International University of Catalunya (UIC), Barcelona, Spain; ^18^ Biomedical Research Networking Centre in Neurodegenerative Diseases (CIBERNED), National Institute of Health Carlos III, Madrid, Madrid, Spain; ^19^ Glenn Biggs Institute for Alzheimer's & Neurodegenerative Diseases, University of Texas Health Science Center at San Antonio, San Antonio, TX, USA; ^20^ Department of Microbiology, Immunology and Molecular Genetics, University of Texas Health Science Center at San Antonio, San Antonio, TX, USA; ^21^ Joe R. and Teresa Lozano Long School of Medicine, University of Texas Health Science Center at San Antonio, San Antonio, TX, USA; ^22^ Clinical Neurochemistry Laboratory, Sahlgrenska University Hospital, Mölndal, Sweden; ^23^ Department of Psychiatry and Neurochemistry, University of Gothenburg, Gothenburg, Sweden; ^24^ Department of Neurodegenerative Disease, UCL Institute of Neurology, London, United Kingdom; ^25^ Hong Kong Center for Neurodegenerative Diseases, Clear Water Bay, Hong Kong, China; ^26^ Wisconsin Alzheimer's Disease Research Center, University of Wisconsin School of Medicine and Public Health, University of Wisconsin‐Madison, Madison, WI, USA; ^27^ UK Dementia Research Institute at UCL, London, United Kingdom; ^28^ Department of Psychiatry and Neurochemistry, Institute of Neuroscience & Physiology, the Sahlgrenska Academy at the University of Gothenburg, Mölndal, Gothenburg, Sweden; ^29^ Neurology Department. Biomedical Research Institute Sant Pau (IIB Sant Pau). Sant Pau Hospital. Universitat Autònoma de Barcelona, Barcelona, Spain; ^30^ Center for Networker Biomedical Research in Neurodegenerative Diseases (CIBERNED), Barcelona, Spain; ^31^ Department of Pathology and Laboratory Medicine, Perelman School of Medicine at the University of Pennsylvania, Philadelphia, PA, USA; ^32^ Department of Neurodegenerative Disease, UCL Queen Square Institute of Neurology, London, United Kingdom; ^33^ Division of Neurosciences, Neurogenetics Laboratory, Center for Applied Medical Research, University of Navarra School of Medicine, Pamplona, Spain; ^34^ CIBERNED, Instituto de Salud Carlos III, Madrid, Spain; ^35^ University of Washington, Seattle, WA, USA; ^36^ Indiana Alzheimer's Disease Research Center, Indiana University School of Medicine, Indianapolis, IN, USA; ^37^ Department of Neurology, Washington University School of Medicine, St. Louis, MO, USA; ^38^ Knight Alzheimer Disease Research Center, Washington University School of Medicine, St. Louis, MO, USA; ^39^ Knight Alzheimer Disease Research Center, St. Louis, MO, USA; ^40^ Saarland University Medical Center, Homburg, Germany; ^41^ Johns Hopkins University School of Medicine, Baltimore, MD, USA; ^42^ Department of Pathology & Laboratory Medicine, Perelman School of Medicine, University of Pennsylvania, Philadelphia, PA, USA; ^43^ Center for Personalized Diagnostics, Hospital of the University of Pennsylvania, Philadelphia, PA, USA; ^44^ Perelman School of Medicine, University of Pennsylvania, Philadelphia, PA, USA; ^45^ Department of Pathology and Laboratory Medicine, Hospital of the University of Pennsylvania, Philadelphia, PA, USA; ^46^ Division of Biostatistics, Washington University in St. Louis, St. Louis, MO, USA; ^47^ Vanderbilt Memory and Alzheimer's Center, Vanderbilt University School of Medicine, Nashville, TN, USA; ^48^ Hope Center for Neurological Disorders, Washington University in St. Louis, St. Louis, MO, USA; ^49^ Washington University School of Medicine, St. Louis, MO, USA

## Abstract

**Background:**

Cerebrospinal fluid (CSF) biomarkers, including amyloid‐β 42 (Aβ42), have emerged as essential endophenotypes in genome‐wide association studies (GWAS) of Alzheimer's disease (AD), advancing our understanding of AD biological processes beyond traditional case‐control studies. Using the largest sample size to date (*N* = 18,491), we aim to elucidate sex‐specific associations with AD pathology by performing sex‐stratified GWAS of three well‐established CSF endophenotypes, Aβ42, Tau, and phosphorylated tau (pTau181).

**Method:**

We conducted meta‐analyses of sex‐stratified GWAS for each CSF biomarker, leveraging 22 US and European cohorts with available raw CSF and genotype data (*N* = 6,785; 51.84% male; age=68), along with summary statistics from six external cohorts (*N* = 11,706; 45.27% male; age=69). Consistent quality control was applied prior to genetic analyses, including z‐score standardization on raw CSF biomarker values in internal cohorts. The GWAS adjusted for age, ten principal components of genetic ancestry, and cohort‐array combination as applicable. We defined a sex‐specific effect as a variant association that reached genome‐wide significance in one sex and had non‐overlapping 95% confidence intervals of the effect estimates between sexes.

**Result:**

We identified seven genome‐wide significant loci, including four previously reported loci and three novel female‐specific associations, including one for Aβ42 (rs372578, p(Females)=1.86E‐08, b(F)=‐0.09, p(Males)=0.78), Figure 1), one for Tau (rs1582763, p(F)=5.56E‐09, b(F)=‐0.09, p(M)=0.05, Figure 2), and one for pTau181 (rs6434518, p(F)=2.95E‐08, b(F)= 0.17, p(M)=0.80, Figure 3). The lead Aβ42 variant, rs372578, is an eQTL for *BMP6* (*p* = 8.00E‐04, http://www.braineac.org), which encodes a TGF‐beta ligand involved in iron homeostasis and bone/fat development. Increased expression of *BMP6* is linked to hippocampal neurogenesis defects in AD patients and APP‐transgenic mice. The lead Tau variant, rs1582763, is in the *MS4* locus, an established genetic risk factor for AD with some evidence of female‐specificity, and has been linked to soluble *TREM2* level regulation in CSF. Finally, the top pTau181 variant, rs6434518, is an eQTL for immune response genes *STAT4, STAT1* (*p* = 2.40E‐02), and *MYO1B* (*p* = 2.60E‐02) involved in lipid metabolism and proteostasis.

**Conclusion:**

Our results highlight significant female‐specific genetic associations across CSF biomarkers, underscoring the importance of sex‐specific genetic analyses in deepening understanding of AD genetic architecture.